# Gene expression profiling reveals candidate biomarkers and probable molecular mechanisms in chronic stress

**DOI:** 10.1080/21655979.2022.2040872

**Published:** 2022-02-19

**Authors:** Bohan Zhang, Weijie Zhong, Biao Yang, Yi Li, Shuxian Duan, Junlong Huang, Yanfei Mao

**Affiliations:** a Department of Anesthesiology and Surgical Intensive Care Unit, Xinhua Hospital, Shanghai Jiaotong University School of Medicine, Shanghai, SH, China; b Department of Neurosurgery, Ninth People Hospital Affiliated to Shanghai Jiao Tong University School of Medicine, Shanghai, SH, China; c Department of Neurosurgery, Huashan Hospital, Fudan University, Shanghai, SH, China

**Keywords:** Biomarkers, mRNA, stress, lung, immune

## Abstract

Chronic stress refers to nonspecific systemic reactions under the over-stimulation of different external and internal factors for a long time. Previous studies confirmed that chronic psychological stress had a negative effect on almost all tissues and organs. We intended to further identify potential gene targets related to the pathogenesis of chronic stress-induced consequences involved in different diseases. In our study, mice in the model group lived under the condition of chronic unpredictable mild stress (CUMS) until they expressed behaviors like depression which were supposed to undergo chronic stress. We applied high-throughput RNA sequencing to assess mRNA expression and obtained transcription profiles in lung tissue from CUMS mice and control mice for analysis. In view of the prediction of high-throughput RNA sequences and bioinformatics software, and mRNA regulatory network was constructed. First, we conducted differentially expressed genes (DEGs) and obtained 282 DEGs between CUMS (group A) and the control model (group B). Then, we conducted functional and pathway enrichment analyses. In general, the function of upregulated regulated DEGs is related to immune and inflammatory responses. PPI network identified several essential genes, of which ten hub genes were related to the T cell receptor signaling pathway. qRT-PCR results verified the regulatory network of mRNA. The expressions of CD28, CD3e, and CD247 increased in mice with CUMS compared with that in control. This illustrated immune pathways are related to the pathological molecular mechanism of chronic stress and may provide information for identifying potential biomarkers and early detection of chronic stress.

## Graphical Abstract

**Figure:**
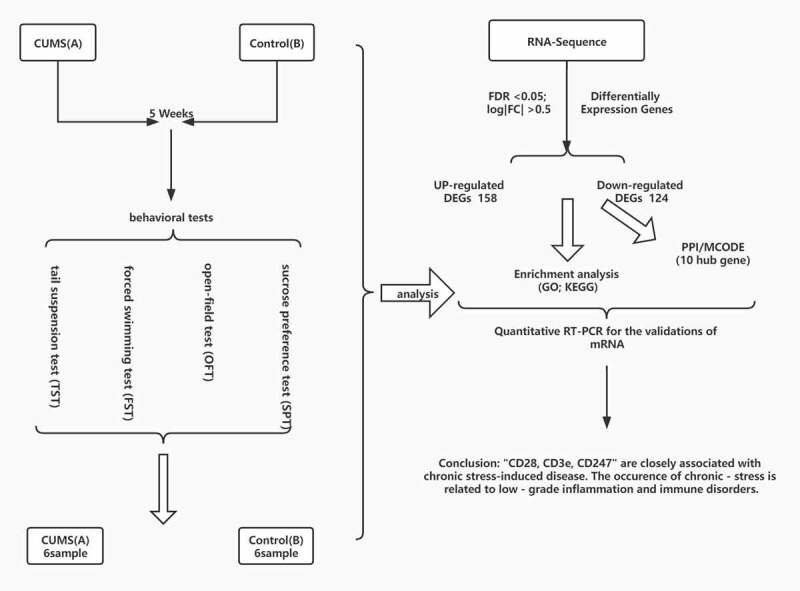

## 1. Induction

Chronic stress refers to nonspecific systemic reactions under the over-stimulation of different external and internal factors for a long time [1]. A stressor may be physical, chemical, or psychological in nature, and chronic psychosocial stress may play a crucial role due to its long-lasting effects [2]. Numerous studies confirmed that chronic psychosocial stress has adverse effects on almost all tissues and organs, including but not limited to metabolic, cardiovascular, immune, and neuronal [3]. During the occurrence and development of many chronic systemic diseases, including Cardiovascular diseases (CVDs), tumors, depression, and Alzheimer’s disease (AD) [^4–7^]. Notably, the physiological response to chronic psychosocial stress also has long been considered as an essential factor in the development of different pulmonary diseases [8], including asthma, chronic obstructive pulmonary disease, and lung cancer [9,10]. The incidence and mortality of chronic psychosocial stress-related diseases, including pulmonary disease, are increasing in people aged over 60 [11]. In the present coronavirus disease 2019 (COVID-19) pandemic, patients under high stress at the time of pulmonary infection have exhibited increased mortality compared to subjects under low levels of stress [12]. Chronic psychosocial stress leads to an adverse effect on the lung and contributes to different diseases. Therefore, identifying the mechanism may be critical to the intervention of chronic stress-related diseases.

Previous studies on mechanisms that chronic stress contributes to chronic systemic diseases have mainly focused on aspects including the hyperactivity of hypothalamic-pituitary-adrenal (HPA), as well as sympathetic adrenal-medullary (SAM) axes [13]. Recent studies have demonstrated that chronic stress negatively influences immune regulation and promotes inflammation, which has been increasingly considered a crucial factor to explain the effect of chronic stress [14]. Chronic stress has been gradually regarded as low-grade chronic inflammation involved with a higher level of proinflammatory cytokines and increased expressions of chemokines, including CCL2, CXCL2, and CXCL3 [15]. As studies reported, chronic stress augments the outputs of monocytes and increases the infiltration of macrophages into tissues like the lung, which provides an underlying mechanism to the progression of lung tumors [16]. What is more, it also compromises NK cell cytotoxicity and suppresses NK cell activity [17]. In addition, under the condition of chronic stress, dendritic cells (DCs) are prevented from maturation in response to inflammatory stimuli. DCs are unable to prime T helper type 1 (Th1) cells efficiently, which results in a significant decrease in the number of effector cells producing interferon -γ (IFN-γ). All above presents that the immuno-harmful effects of chronic stress may regulate local and systemic inflammatory mediator production. According to previous study, immuno-harmful effects and inflammatory mediator production also play an important role in lung injury, which always included the release of chemokine and proinflammatory cytokines (TNF-α, IL-6, IL-8 and IL-1β) [9]. The immune response of immune cells (including T cells, NK cells, macrophages, etc.) is an important factor in the generation and development of lung injury [18]. Consequently, the molecular changes related to immune and inflammatory pathways are worthy of attention and study.

CUMS(chronic unpredictable mild stress) has already been a traditional model for numerous studies [19]. The CUMS model was usually applied to study depression [20]. It was also used to study the effects of chronic stress on the occurrence, development, and aggravation of colonic inflammation, gastric precancerous lesions, and other systemic diseases [21,22]. As a result, in the experiment, we adopt CUMS model for studying chronic stress.

The association between immune factors and chronic stress has gradually been the focus of research. Although numerous studies have noted the relationship between chronic stress and geometric features, few obtained accordant results to identify the progress of chronic stress. In our study, we determined to search out the potential genes involved in the pathogenesis of chronic stress by gene expression profiling analysis. We assumed that the genes might provide new molecular biomarkers and identify the molecular mechanism of the systemic responses to chronic stress, which may help determine pathophysiological pathways and potential disease-related mechanisms involved in systemic consequences of chronic stress. Our work may help to study further the pathogenesis of chronic stress-induced consequences, especially the influence on different lung diseases.

## 2. Materials and methods

### 2.1. Animals

We purchased male BALb/c mice (aged 4 weeks weighted 20 ± 2 g) from Shanghai Jihui Laboratory Animal Care Co, Ltd. (Shanghai, China). We fed the mice in the Shanghai Xinhua Hospital animal laboratory under a specific pathogen-free environment. Mice were housed in groups of 3 mice per cage, The humidity was 40–60%, and the temperature was kept at 24 ± 2°C. The mice were provided with standard water and food. We carried out all the experiments under the rules approved by the Shanghai Xinhua Hospital Ethics Committee (no. XHEC-NSFC-2020-049).

### 2.2. Chronic unpredictable mild stress (CUMS) procedure

Mice adapted to the environment for at least one week before use (N = 6 in each group). The stressed mice were housed in a different room in contrast to the control group. The Chronic unpredictable mild stress (CUMS) procedure was conducted as published [23,24]. Experimental mice were divided into two groups. The control group was kept in an undisturbed cage. For CUMS, we used a method of stress intervention in mice at a different time, with a total of 7 kinds of random sort of stress methods, including tilted cage, damp bedding, confinement in the tube for 2h, 5 minutes of 45°C oven, cage shaking for 10 minutes, swimming for 5 minutes at 4°C, exposure to empty bottles, food and water deprivation [23]. The procedure of CUMS is listed in Table 1. After stress five weeks, we begin the animal behavioral experiments and then gather the animal tissues. We anesthetized the mice with 5% isoflurane in oxygen in a plexiglass cage. After anesthesia, mice were sacrificed and lung tissue samples were extracted and rinsed in cold PBS. Then the collected lung tissue was stored at −80°C.

**Table 1.** Chronic unpredictable mild stress (CUMS) procedure

Week	Monday	Tuesday	Wednesday	Thursday	Friday	Saturday	Sunday
First Week	8:00am – 10:00pm tilted cage	15:30 pm – 17:30 pm confinement in tube for 2 h	7:00 pm – next day damp bedding	10:00 am – 12:00 am cage shaking for 10 min	9:00 am – 11:00 am swimming at 4°C for 5 min	9:00 am – 11:00 am 45°C oven for 5 min	7:00 pm – next day space reduction
Second Week	15:30 pm – 17:30 pm confinement in tube for 2 h	8:00am – 10:00pm tilted cage	10:00 am – 12:00 am cage shaking for 10 min	7:00 pm – next day damp bedding	9:00 am – 11:00 am 45°C oven for 5 min	9:00 am – 11:00 am swimming at 4°C for 5 min	2:00 pm – next day food and water deprivation
Third Week	7:00 pm – next day space reduction	7:00 pm – next day damp bedding	9:00 am – 11:00 am swimming at 4°C for 5 min	8:00am – 10:00pm tilted cage	9:00 am – 11:00 am exposure to empty bottles	15:30 pm – 17:30 pm confinement in tube for 2 h	2:00 pm – next day food and water deprivation
Fourth Week	9:00 am – 11:00 am 45°C oven for 5 min	9:00 am – 11:00 am exposure to empty bottles	15:30 pm – 17:30 pm confinement in tube for 2 h	7:00 pm – next day space reduction	10:00 am – 12:00 am cage shaking for 10 min	2:00 pm – next day food and water deprivation	8:00am – 10:00pm tilted cage
Fifth Week	15:30 pm – 17:30 pm confinement in tube for 2 h	9:00 am – 11:00 am swimming at 4°C for 5 min	7:00 pm – next day space reduction	9:00 am – 11:00 am exposure to empty bottles	2:00 pm – next day food and water deprivation	7:00 pm – next day damp bedding	9:00 am – 11:00 am 45°C oven for 5 min

### 2.3. Behavioral tests

We conducted the test in dim light during the daylight phase (from 9 a.m.to 3 p.m.), and the test included tail suspension test (TST), spontaneous activities, and forced swimming test (FST). The tests were performed as reported in previous articles [25,26]. For the sucrose preference test, one day after the last stress session, mice have no access to food and water for 24 h during the test. After deprivation, all animals are allowed to have food and water for 12 h, one tube of regular water, and one tube of 1% (wt/vol) sucrose solution. The weight of each tube was recorded before and after the test. After the test, all animals were sent back for group housing. Finally, we calculated the reduction and acquired a sucrose preference ratio [27]. For spontaneous activities, one day after the last stress session, we use OFT (open-field test). Mice were allowed to explore for 5 min after entering into the testing box(50 × 50 × 40 cm). The time in the center area and total distance were tracked and measured by software (Shanghai Jiliang Software Technology Company, Shanghai, China). For the forced swimming test (FST), three days after the last stress session, mice were put in a water tank (25cmhigh,10 cm in diameter, filled with24 ± 1°C water) for 6 min, and the time of immobility was recorded with in the last 4 min of the test. The immobility time was analyzed by Jiliang software (Shanghai Jiliang Software Technology Company, Shanghai, China). For the tail suspension test (TST), two days after the last stress session, each mouse was suspended by the end of their tail, 50 cm above the floor) in a sound-isolated room. The suspension of mice was tracked for 6 minutes, and the last four-minute suspension was recorded as the time of immobility time by two trained investigators who were unaware of the strain in a blinded manner using Jiliang software (Shanghai Jiliang Software Technology Company, Shanghai, China) [23]. We performed the behavioral test to assess whether the stimulation amount of CUMS reached the threshold to cause depression-like behavior and inflammation.

### 2.4. RNA isolation and library preparation

We extracted 50 mg of tissue from each sample (n = 6 per group). RNA-Seq technology (OE Biotech, Shanghai, China) was adapted to analyze the transcriptome genes expression. Agilent 2100 Bioanalyzer (Agilent Technologies, Santa Clara, CA, USA) was applied to estimate the integrity of RNA. TruSeq Stranded mRNA LT Sample Prep Kit (Illumina, San Diego, CA, USA) was used to construct the libraries following the manufacturer’s protocol.

### 2.5. RNA sequencing and differentially expressed genes analysis

The library was sequenced on the Illumina HiSeq X Ten platform. 150 bp paired-end reads were generated. Firstly, we used Trimmomatic to process raw data (raw reads) of fastq format [28]. To obtain the clean reads, the low-quality reads were removed. The clean reads were mapped to the human genome (GRCh38) using HISAT2 [29], and through HTSeqcount, we obtained the read counts of each gene [30]. Differential expression analysis was performed using the DESeq (2012) R package [31]. Hierarchical clustering is presented through volcano plots and heatmaps (http://www.bioinformatics.com.cn/).

### 2.6. Functional analysis and PPI analysis

To investigate the molecular biological processes involved in CUMS-induced chronic stress model, we submitted DEGs to DAVID (2021 Update) for GO and KEGG functional analysis [32]. KEGG pathway and GO terms molecular functions(MF), biological processes(BP), and cellular components(CC) with P < 0.05 and counts > 2 were considered significantly enriched by the common DEGs. In order to find the essential genes related to the CUMS model and analyze them at the mRNA level, we used [33] STRING (http://string-db.org) to predict the interaction between DEGs and the functional proteins encoding proteins and designed a confidence score of >0.70. Then, Cytoscape3.9.0 was used to construct a PPI network diagram, and the PPI network was calculated and visualized. Cytoscape3.9.0 was used to beautify the PPI network diagram and calculate the degree of connectivity, which is an important parameter. A high degree of connectivity indicates that proteins interact with more peripheral proteins and play more important roles. And it is believed that the genes with higher connectivity are relatively vital in the whole network diagram [34]. Connectivity degree analysis was performed, and the most highly connected cluster was extracted from the PPI network through MCODE analysis [35].

### 2.7. Quantitative RT-PCR techniques

qRT-PCR was applied to qualify the RNA sequencing data, and we detected ten mRNAs that were significantly different between the control and the CUMS mice (n = 6/group). Firstly, the RNAiso Plus Kit (TaKaRa) was applied to extract total RNA from the lung. After that, we used PrimeScript™ RT reagent Kit (TaKaRa), and RNA was reverse-transcribed into cDNA. qRT-PCR was carried out through SYBR® Premix Ex Taq™ kit (TaKaRa) on biosystems QuantStudio 5 Flex (Thermo Fisher Scientific, Waltham, MA, USA) according to the manufacturer’s instruction (all gene primers are listed in Table 2). The relative expression level of mRNAs in the lung was compared to actin. We chose the 2^−ΔΔCt^ method to calculate the results. Each sample of qRT-PCR was repeated in three technical replications.

**Table 2.** Gene primer sequence

GENE	Primer sequence
Lck_	Forward TTTGAGAAGGGTGAACAGC
	Reverse AGTTGAAGGGAATGAAGCC
Cd3e_	Forward GGTCCTGCCCCATTTATAG
	Reverse GCCTTTTGCATTAGCAGAG
Cd8a	Forward ACCCGAACTCCGAATCTT
	Reverse AGAGCATCCTTGCGAAAC
CD3g	Forward ACTGTAGCCCAGACAAATAAAGC
	Reverse TGCCCAGATTCCATGTGTTTT
CD247	Forward TTCAGAACTCACAAGGACCCT
	Reverse GCTACTCTGCTGGGTGCTTTC
CD28	Forward GTTCTTGGCTCTCAACTTCTTCT
	Reverse TGAGGCTGACCTCGTTGCTAT
CD4	Forward AGGTGATGGGACCTACCTCTC
	Reverse GGGGCCACCACTTGAACTAC
Cd3d	Forward TCCTGAAATCTCCCTCTGG
	Reverse TGCATGACGCTGGTATTG
Prkcq_	Forward TCGGGGTGCTCGTTTAT
	Reverse TCGGGTAGAAGGGGTTG
itk_	Forward CGCTACTACGTGGCTGAGA
	Reverse AGCGGAGTCGAGTGACC

## 3. Result

In our study, we determined to search out the potential genes involved in the pathogenesis of chronic stress by gene expression profiling analysis. We assumed that the genes might provide new molecular biomarkers and identify the molecular mechanism of the systemic responses to chronic stress, which may help determine pathophysiological pathways and potential disease-related mechanisms involved in systemic consequences of chronic stress. We conducted differentially expressed genes (DEGs) and obtained 282 DEGs between CUMS (group A) and the control model (group B). Then, we conducted functional and pathway enrichment analyses. In general, the function of upregulated DEGs is related to immune and inflammatory responses. PPI network identified several essential genes, of which ten hub genes were related to the T cell receptor signaling pathway. qRT-PCR results verified the regulatory network of mRNA.

### 3.1. Behavioral tests

**Figure 1.**
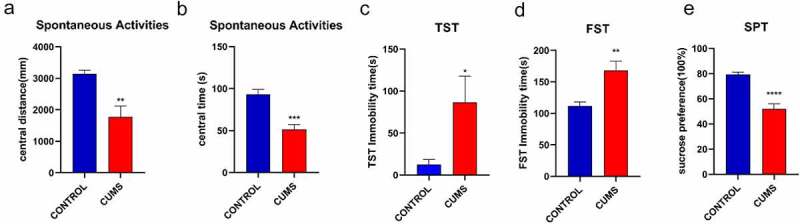
CUMS leads the mice to express depression-like behaviors. Mice were subjected to the adaptation for a week, the CUMS for 5 weeks. (a) The values of central distance in the Spontaneous Activities were 1766.91 ± 349.1 mm in CUMS-treated mice (n = 12) and 3140.31 ± 115.6 mm in controls (P < 0.01). (b) The values of central time in the Spontaneous Activities were 51.32 ± 6.2 seconds in CUMS-treated mice and 93.26 ± 5.5 seconds in controls (P < 0.001). (c) The values of immobility time in TST were 86.42 ± 31.3 seconds in CUMS-treated mice (n = 12) and 12.28 ± 6.3 seconds in control mice (P < 0.05). (d)The values of immobility time in FST were 168.30 ± 14.4 seconds in CUMS-treated mice (n = 12) and 111.67 ± 6.3 seconds in control mice (P < 0.01). (e) The values of SPT is 51.87 ± 4.2 seconds in CUMS-treated mice and 79.41 ± 1.7 seconds in controls (P < 0.0001). The results are expressed as mean ± SEM. n = 12 per group, ***P < 0.001 ****P < 0.0001 compared with control.

**Abbreviations**: CUMS, chronic unpredictable mild stress; FST, forced swimming test; TST, tail suspension test; SPT, sucrose preference test; SEM, standard error of the mean.

The sign of successful induction of CUMS tested Spontaneous Activities, TST, FST, SFT. In the Spontaneous Activities, the time and distance of the stressed mice in the center were significantly reduced (Figure 1(a,b)). The CUMS mice also showed higher immobility in TST (Figure 1(c)), and FST (Figure 1(d)) compared to the control group. The result indicated that we obtained depression-like mice. In addition, animals that are successfully modeled by CUMS will show a significant reduction in sucrose preference (Figure 1(e)). Our data illustrate the mice in the model group showed depression-like behavior, which meant the stimulation of CUMS procedure attained to a certain extent.

### 3.2. RNA sequencing and differentially expressed genes analysis

**Figure 2.**
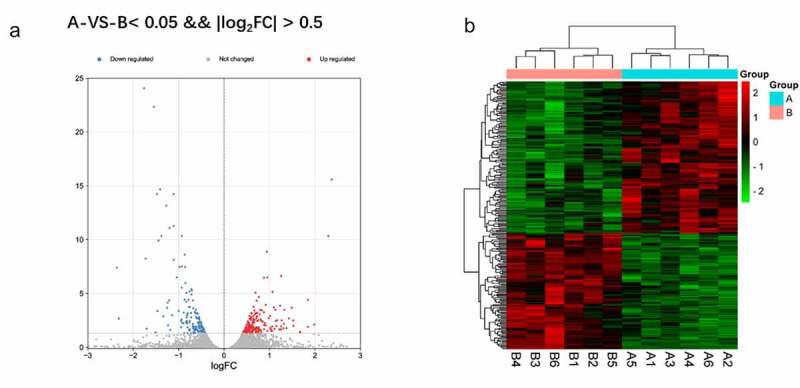
a Reflect the difference caused by the comparison in the volcano map, gray is the genes with insignificant differences, and red and blue are genes with significant differences. The horizontal axis is log2FoldChange, and the vertical axis is – log10FDR. b The red is the figure indicates the relatively high expression protein coding gene, and the green indicates the relatively low expression protein coding gene.

A total of 282 DEGs between CUMS (group A) and normal model (group B) were obtained with the criteria of log|FC| >0.5 and FDR <0.05. In the heat map and the volcano plot, the 158 upregulated genes and the 124 down-regulated genes were shown (Figure 2(a,b))

### 3.3. Functional analysis

**Figure 3.**
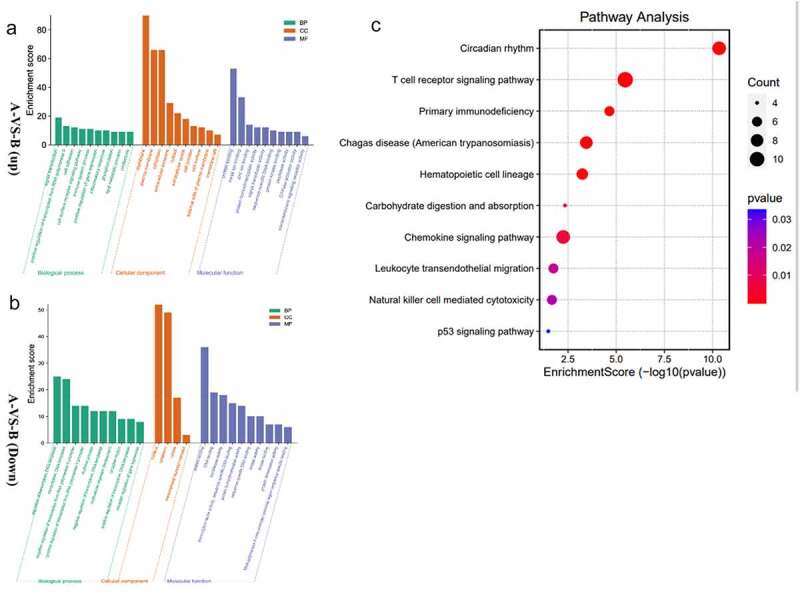
a/b The horizontal axis is the name of GO item, and the vertical axis is log10pValue. **c** The horizontal axis of the figure is the enrichment score. The larger the bubble, the more the number of differential protein encoding gene. The bubble color changes from red-purple-blue, the smaller the enrichment p Value, the greater the significance.

To reveal further the biological function of DEGs, we used the DAVID online tool for functional enrichment analysis of DEGs. For GO analysis, we analyzed three functional groups: MF, CC, and BP. The GO analysis results are the GO terms of upregulated DEGs and down-regulated DEGs sorted by p-value (Figure 3(a,b)). In BP, the up-regulation of DEGs is mainly related to the immune system process and inflammatory response. Meanwhile, the down-regulation of DEGs is mainly related to the rhythmic process. In CC, the up-regulation of DEGs was mainly in the extracellular space plasma membrane external side of the plasma membrane. The down-regulation DEGs are concentrated in the nucleus, cytoplasm, and cytosol. As the MF analysis showed, most of the up-regulation of DEGs was associated with protein binding. The result also showed that the down-regulation of DEGs has a connection with transferase activity kinase activity. Analyzing the KEGG pathway, we found that the DEGs were mainly enriched in the T cell receptor pathway and primary immunodeficiency. (Figure 3(c)).

In general, the function of upregulated DEGs is related to immune and inflammatory responses distributed in the plasma membrane.

### 3.4. PPI analysis

**Figure 4.**
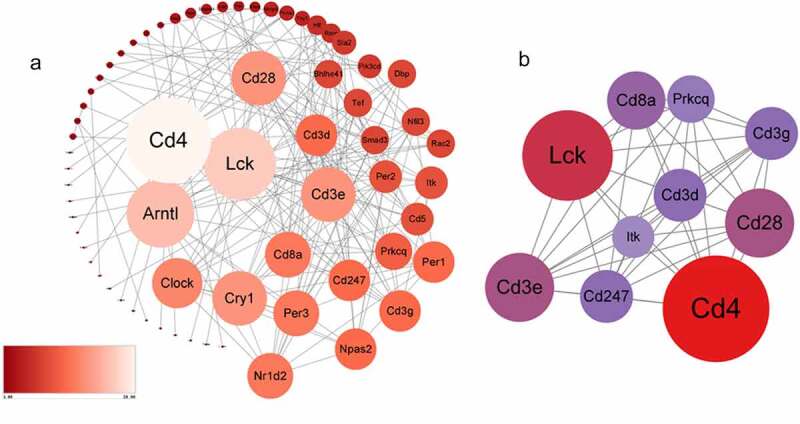
a The protein-protein interaction network consists of 66 nodes and 176 edges. Color and size represent the connectivity degree of nodes. b The most highly connected cluster is composed of ten ‘Lck, Cd3e, Cd4, Cd3g, Cd3d, Cd8a, Prkcq, itk,’ Cd28, Cd247 hub genes.

282 DEGs were submitted to the STRING to predict protein interactions. When the interaction score was >0.700, the PPI network consisted of 66 nodes and 176 edges (Figure 4(a)). The degree of connection is an important factor. High connectivity means that the protein has higher interaction with surrounding proteins and plays an important role. The tighter the connection, the larger the node area. Larger sizes of the nodes show higher connectivity degree, which was marked with Light blue–yellow – red. Subsequently, we analyzed the PPI network and extracted the most highly connected cluster by MCODE plug-in in Cytoscape (Figure 4(b)). In the top 10 connectivity degree genes, we find that all of them ‘Lck, Cd3e, Cd4, Cd3g, Cd3d, Cd8a, Prkcq, itk, Cd28, Cd247’ are related to the T cell receptor pathway and immune process. According to past research, both immune and inflammation are essential molecular for chronic stress.

### 3.5. Quantitative RT-PCR for the validations of mRNA

**Figure 5.**
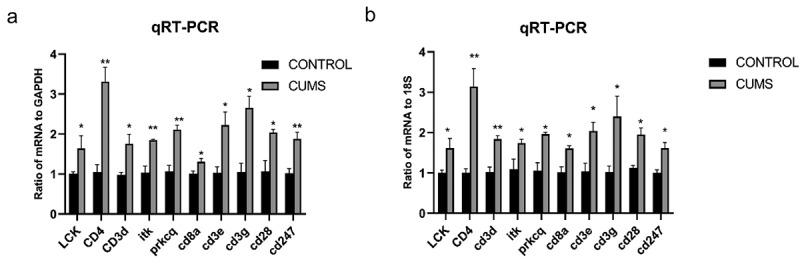
The validation of differentially expressed mRNAs in the lung tissue from mice with CUMS-induced depression-like behaviors and controls. **Notes**: Ten mRNAs were involved in immue pathways and were selected for qRT-PCR analysis(a) qRT-PCR was used to analyze the relative values of Lck, Cd3e, Cd4, Cd3g, Cd3d, Cd8a, Prkcq, itk, Cd28, Cd247 from mice with CUMS-induced depression-like behaviors and controls (n = 6 per group), in which GAPDH was used for endogenous control (b) The relative level of of Lck, Cd3e, Cd4, Cd3g, Cd3d, Cd8a, Prkcq, itk, Cd28, Cd247 gene expression from mice with CUMS-induced depression like behaviors (n = 6) and controls (n = 6), which were analyzed using 18s as the internal control. The relative values for control mice were normalized to be one. The data are expressed as mean ± SEM. *P < 0.1, **P < 0.01.

**Abbreviations**: CUMS, chronic unpredictable mild stress; SEM, standard error of the mean

In order to verify the DEGs, we selected three significantly changed mRNAs. We performed qRT-PCR from the lung tissues, which was used for mRNA sequencing. The expressions of Lck, Cd3e, Cd4, Cd3g, Cd3d, Cd8a, Prkcq, itk, Cd28, Cd247 were increased in mice with CUMS compared with that in control using GAPDH for endogenous control (Figure 5(a)).and 18s for endogenous control (Figure 5(b)). The results of qRT-PCR are significant support to demonstrate our research.

### 3.6. Statistical analysis

All data were analyzed with the statistical program SPSS17.0 (Chicago, IL, USA). Data are expressed as means ±SEM. Statistical comparisons between the experimental group and control group were performed using a two-sample t-test (T-T test) with an additional Bonferroni post hoc test. P < 0.05 was considered statistically significant.

## 4. Discussion

Stress can be divided into acute stress and chronic stress, but the effects of short-term and long-term stress on the body are not totally the same and are worthy of study. The ‘fight‐or‐flight’ response of acute stress allows us to act in a way to prevent injury and provide energy to the body, which is considered a protective effect [36]. Chronic psychological stress often aggravates some diseases such as pneumonia and some other diseases by increasing the levels of chemokines and inflammatory factors [9]. In this process, the activation of immune cells, including NK cells and T cells, also played an important role [37]. In previous studies of our group, it was also found that when the procedure of unpredictable stress exceeds a certain number of days, the body presents high levels of inflammatory factors, including IL1β, IL-6, and TNFα. Before this, these indicators showed a lower level in the stress group than that in the control group (Data was not shown here).

Moreover, determining the molecular targets for chronic stress is necessary for targeting the detrimental consequences of chronic stress. It will help identify the progression associated with pulmonary and other diseases. Therefore, we investigated the DEGs of CUMS according to the PPI network analysis. A total of 282 DEGs were screened, of which 158 genes were upregulated, and 124 were downregulated. Then we identify ten ‘key genes’ (Lck, Cd3e, Cd4, Cd3g, Cd3d, Cd8a, Prkcq, itk, Cd28, Cd247). These ten genes demonstrated a high connection with chronic stress. The inline results from sequencing mRNAs, as well as qRT-PCR, fortify our results. Notably, ‘Lck, Cd3e, Cd4, Cd3g, Cd3d, Cd8a, Prkcq, itk, Cd28, Cd247’ are related to the T cell receptor pathway and immune process. Moreover, our results of dual-luciferase reporter assay and qRT-PCR also enhance the bioinformatics analysis about targeted therapeutics. According to the research report, the significance of immune has been highlighted in chronic stress [15]. The association between immune factors and chronic stress has become a focus of research. However, there are no clear biomarkers to identify the progress of chronic stress. Our research indicated that immune progress is closely related to chronic stress. Furthermore, the upregulated DEGs ‘Lck, Cd3e, Cd4, Cd3g, Cd3d, Cd8a, Prkcq, itk, Cd28, Cd247’ were mainly in the center of the network as the PPI network analysis showed. Interestingly, all of them are related to immune progress. In the above, our results highlighted the key genes found in the sub-networks (‘Cd28’, ‘Cd3e’, ‘CD247’) that play an essential role in the processes of chronic stress.

CD28 is a fundamental member of a subfamily of co-stimulatory molecules which encode an extracellular variable immunoglobulin-like domain [38]. As a major costimulatory receptor, CD28 is a key regulator of immune responses [39]. Every adaptive immune response requires co-stimulation through the B7/CD28 co-stimulatory axis [40]. CD28 is also essential for promoting the proliferation and effector function of conventional T cells [41]. T cell activation has a key role in the development of inflammation [42,43]. Full activation of T cells requires at least two signals. Signal one requires the antigen major histocompatibility complex protein present on the surface of antigen-presenting cells to bind to T cell receptor [44], while signal two requires the participation of CD28 [45]. In Staphylococcus aureus and Streptococcus-induced pneumonia, CD28 directly binds to superantigens as a direct receptor for superantigen toxins [46]. T cells activated by a second signal release numerous inflammatory cytokines, including IL-2, IFN-γ, and TNF, that induce toxic shock [47]. Subsequently, a lethal cytokine storm leading to lethal shock is triggered after overstimulating T cell-mediated immune [48]. In addition, CD28 and its relating inducible co-stimulator (ICOS) signal mediate T cell cytoskeleton remodeling, cytokine production, and enhancement [38,49]. In systemic sclerosis-induced pulmonary fibrosis, the CD28-ICOS signal is involved in Th2 effector cell differentiation in the lung [50]. Moreover, CD28 among with ICOS promotes the differentiation of naive CD4 T cells into effector T helper cells producing IL-17, and the overexpression of IL-17 in lung epithelial cells leads to chemokine production and leukocyte infiltration, resulting in lung injury [51]. Additionally, the CD-28-ICOS signal also plays an important role in the pathogenesis of community-acquired pneumonia initiated by Staphylococcus aureus and pneumonia induced by the airway pathogens, including Klebsiella pneumonia, Pseudomonas aeruginosa, and Streptococcus pneumonia [52]. As mentioned above, it is known that CD28 acts as an essential role in bacterial pneumonia, lethal shock, and pulmonary fibrosis in autoimmune diseases. Our result suggested that CD28 was significantly upregulated in chronic stress. Whether it can be used as a potential target for chronic stress and the mechanisms are what we need to study further.

CD3e is one of the multiple signaling subunits of T-cell antigen receptor (TCR) [38]. CD3 molecules bind to the T cell receptor (TCR) and form the CD3/TCR complex, which mediates TCR signaling and T cell differentiation [39,40]. CD3ε, encoded by the (CD3e) gene, is associated with severe immune deficiency and is frequently used as a protein target for CD3 antibodies [41,42]. Previous studies illustrated that CD3e is associated with intestinal inflammation [43]. CD3ε was also reported related to Type 1 diabetes (T1DM). T1DM is an autoimmune disease in which the immune system mistakenly targets and destroys pancreatic beta cells [44]. Studies demonstrated that alternative T-cell-targeted therapies targeting CD3ɛ could alter the course of T1DM through downregulating of CD3ε/TCR signal and inhibiting the proliferation of T lymphocytes [45]. CD3e was found to potentially serve as an indicator of tumor microenvironment (TME) regulation in bladder cancer, and the therapies targeting CD3e can be novel therapeutic strategies [46]. CD3e has also been shown to be involved in the development of asthma. In CD3e+ differentiated T cells, especially those producing IL-4 and IL-5, CCL21-CCR7 signaling was inhibited, which led to increased airway resistance and aggravated lung injury [47]. Moreover, CD3e is one of the top prognostic factors for the analysis of host genetic data for SARS-CoV-2, which illustrates its prognostic value in COVID-19 development [48]. Interestingly, our result suggested CD3e was significantly upregulated. Therefore, the role CD3e plays in chronic stress and the underlying mechanisms are what we determine to study.

CD247, also known as the T cell surface glycoprotein CD3 zeta chain, is part of the T cell antigen receptor (TCR) complex and plays an important role in receptor expression and signaling [49]. Abnormalities in this pathway can lead to T cell dysfunction and the development of autoimmune diseases [50]. In chronic inflammatory diseases, the abnormal expression of CD247 is associated with altered T cell activity [51,52]. The abnormal regulation of CD247 has been reported in chronic inflammatory diseases such as celiac disease [53], chronic obstructive pulmonary disease [52], systemic lupus erythematosus [54], and systemic sclerosis [55]. Studies have shown that abnormalities in CD247 are associated with chronic inflammation-induced immune disorders, including chronic infections (HIV, hepatitis C, and leprosy) and autoimmune diseases (arthritis, contact eczema, and lupus) [51]. Therefore, it is proposed as a biomarker to assess the immune status of patients with pathologies characterized by chronic inflammation. It has been suggested that the expression levels of CD247 may be a sensitive and predictive biomarker of chronic inflammation in diabetes [56]. Notably, according to the Lung MAP database, CD247 is mainly expressed by T cells and NK cells in the human lung, and there exists a significant correlation between genes and lung function [57], suggesting that CD247 may be an important regulator of immune responses in the lung [58]. In addition, studies have pointed out that CD247 can be used as a potential biomarker for evaluating the severity of T cell-derived disease in patients with idiopathic pulmonary fibrosis [59]. In previous studies, CD247 in sepsis is also enriched in the T cell pathway and is related to the development and prognosis of sepsis [60]. Taken together, CD247 plays a crucial role in inmmuo-inflammation systematic diseases. However, the function and effect of CD247 in the development of chronic stress have not been demonstrated clearly. However, our result suggested that CD247 was significantly upregulated, and the mechanisms are valuable to study in further research.

In summary, these genes are related to immune and inflammatory responses and might be a potential molecular biomarker. Further experimental verification is needed to determine whether these genes are involved in stress-induced disease development and to determine specific underlying mechanisms. However, there were several limitations in the present study, such as a small sample size and lack of further verification tests.

## 5. Conclusion

All above, we finished a series of bioinformatics analyses for RNA sequencing data gained from the lung tissue of the CUMS mice. We aimed to find the potential mechanism for chronic stress that negatively affects almost all tissues and organs. Interestingly, we find that CD28, CD3e, CD247 are closely associated with the chronic stress-induced disease. Our study indicated that the low-grade inflammation caused by immune disorders might be a potential mechanism of the development of stress-induced disease and provide a new perspective to determine the developmental stage of chronic stress. It should be emphasized that a certain threshold amount of stimulation can cause serious consequences, and it is necessary to avoid the stimulation amount reaching the threshold. In addition, there are still limitations. Therefore, the next step of our study is to analyze the critical genes in clinical patients with chronic stress and investigate the experimental validation with clinical information.

## Supplementary Material

Supplemental Material
