# Supplementary material for: Gene expression profiling reveals candidate biomarkers and probable molecular mechanisms in chronic stress
Source: Bioengineered. 2022 Feb 19;13(3):6048–60. doi: 10.1080/21655979.2022.2040872 (PMC8973686; doi:10.1080/21655979.2022.2040872)
Supplement: Supplemental Material [file KBIE_A_2040872_SM6012.docx]

**Genome Annotation File**

The genome annotation file is as follows:

<ftp://ftp.ncbi.nlm.nih.gov/genomes/all/GCF/000/001/635/GCF_000001635.26_GRCm38.p6/GCF_000001635.26_GRCm38.p6_genomic.gff.gz>

It should be noted here that we cannot upload this type of file when we submit our material. Therefore, we put a link here, you can click the link to download the file.
